# The Human Impact of Earthquakes: a Historical Review of Events 1980-2009 and Systematic Literature Review

**DOI:** 10.1371/currents.dis.67bd14fe457f1db0b5433a8ee20fb833

**Published:** 2013-04-16

**Authors:** Shannon Doocy, Amy Daniels, Catherine Packer, Anna Dick, Thomas D. Kirsch

**Affiliations:** Johns Hopkins Bloomberg School of Public Health, Baltimore, Maryland, United States; Johns Hopkins Bloomberg School of Public Health, Baltimore, Maryland, United States; Johns Hopkins Bloomberg School of Public Health, Baltimore, Maryland, United States; Johns Hopkins Bloomberg School of Public Health, Baltimore, Maryland, United States; Johns Hopkins University School of Medicine and Bloomberg School of Public Health, Baltimore, Maryland, United States

## Abstract

Introduction. Population growth and increasing urbanization in earthquake-prone areas suggest that earthquake impacts on human populations will increase in the coming decades. Recent large earthquakes affecting large populations in Japan, Haiti, Chile and New Zealand are evidence of this trend and also illustrate significant variations in outcomes such damage and mortality levels. The objectives of this review were to describe the impact of earthquakes on human populations in terms of mortality, injury and displacement and, to the extent possible, identify risk factors associated with these outcomes. This is one of five reviews on the human impact of natural disasters. Methods. Data on the impact of earthquakes were compiled using two methods, a historical review from 1980 to mid 2009 of earthquake events from multiple databases and a systematic literature review of publications, ending in October 2012. Analysis included descriptive statistics and bivariate tests for associations between earthquake mortality and characteristics using STATA 11. Findings. From 1980 through 2009, there were a total of 372,634 deaths (range 314,634-412,599), 995,219 injuries (range: 845,345-1,145,093), and more than 61 million people affected by earthquakes, and mortality was greatest in Asia. Inconsistent reporting across data sources suggests that the numbers injured and affected are likely underestimates. Findings from a systematic review of the literature indicate that the primary cause of earthquake-related death was trauma due to building collapse and, the very young and the elderly were at increased mortality risk, while gender was not consistently associated with mortality risk. Conclusions. Strategies to mitigate the impact of future earthquakes should include improvements to the built environment and a focus on populations most vulnerable to mortality and injury.

## Introduction

Earthquakes were responsible for an estimated 1.87 million deaths in the 20^th^ century with an average of 2,052 fatalities per event affecting humans between 1990 and 2010 1 ^,^ 2. The magnitude 8.9 Japan earthquake and resulting tsunami in March 2011 was responsible for more than 28,000 deaths; in comparison, the smaller magnitude 7.0 earthquake occurring in Haiti in January 2010 resulted in an estimated 222,500 fatalities 2. In recent history, the Pacific Rim is the most affected by seismic activity, with 81% of the world's largest earthquakes occurring in this region 3.

Earthquakes result from sudden energy releases in the earth’s crust, which create seismic waves that result in ground shaking. Earthquakes are usually caused by slippage on a fault due to built up friction between tectonic plates but can also be caused by volcanic eruptions or manmade explosions 4. Millions of earthquakes occur each year, though only a small proportion is strong enough to be felt and even fewer cause damage. Earthquakes occur at focal depths of 700 km to just under the earth's surface, and the strength of shaking diminishes with increasing distance from the earthquake's source 5. Earthquake magnitude measures the energy released by an earthquake and is described by the moment magnitude scale, which is a logarithmic scale, so that a magnitude 5 earthquake is about 10 times less powerful than a 6, and 100 times less that a magnitude 7. A magnitude 2.5 earthquake is not generally felt by humans, whereas earthquakes with magnitude >7.0 may cause widespread destruction 6. Earthquake impact is assessed by the Modified Mercalli Intensity Scale, which describes the severity of damages from the event on a scale from I to XII, with I being no damage and XII being complete destruction with no surviving structures. Building design, geography and development indicators are important factors in earthquake vulnerability. The objectives of this review were to describe the impact of earthquakes on the human population, in terms of mortality, injury, and displacement and to identify country and event characteristics factors associated with these outcomes. This is one of five reviews on the human impacts of natural disasters, the others being volcanoes, floods, tsunamis, and cyclones.

## Methods

The impact of earthquakes events was summarized using two methods, a historical review of earthquake events, and a systematic literature review for publications relating to the human impacts of earthquakes with a focus on mortality, injury, and displacement.

**Historical Event Review**

A historical database of significant earthquakes between 1980 and 2009 was created. Four publically available data sources were used to create the most complete possible listing of events, allow for inclusion of both human and geophysical factors, and enable cross checking. The two primary sources were the Centre for Research on the Epidemiology of Disasters International Disaster Database (CRED EM-DAT)^7^ and the National Oceanic and Atmospheric Administration’s National Geophysical Data Center (NOAA-NGDC) Significant Earthquakes Database 8. Earthquakes included in EM-DAT met one or more of the following criteria: ≥10 deaths; ≥ 100 affected; declaration of a state of emergency; or a call for international assistance. Earthquakes included in the NOAA-NGDC database met one of the following criteria: ≥10 deaths; moderate damage (US$ 1 million or more); magnitude ≥7.5; Modified Mercalli Intensity X or greater; or the earthquake generated a tsunami. All events reported by EM-DAT were retained (n=706), and zeroes were converted to missing values for injury, homeless, and affected measures; for deaths and total affected, zeroes were converted to missing values only when no other information was reported. Earthquakes from the NOAA-NGDC database were retained if one of the following criteria were satisfied: magnitude ≥5.5; ≥10 deaths; or ≥100 injured (n=579).

Two additional sources, the United States Geological Survey (USGS) Earthquake Hazards Program Global Database 9 and the Northern California Earthquake Data Center (NCEDC)^10^ were used to collect information on specific earthquake characteristics (coordinates, magnitude, focal depth, additional information when available). When available data from these sources were added for events reported by EM-DAT and/or NOAA-NGDC; new events were added only when mortality was reported by USGS. Earthquakes occurring in uninhabited areas that did not cause injury or death were removed. The final list comprised 953 earthquakes occurring between 1980 and 2009; information on mortality, injury or displacement was reported by one or more sources in 738 events. See http://www.jhsph.edu/refugee/publications_tools/index.html for the database of earthquake events.

The following outcome categories were used to assess risk factors for earthquake-related mortality: no deaths (0 deaths); low (1-9 deaths); medium (10-99 deaths); and high (≥100 deaths). Bivariate tests for associations between mortality and the following characteristics were performed using χ^2^ (categorical measures) and ANOVA (continuous measures): decade, World Health Organization (WHO) region, World Bank income level, gross domestic product (GDP), GINI (measure of income inequality), focal depth, and magnitude. All covariates were significantly associated with earthquake mortality in the univariate analysis and were subsequently included in a multinomial logistic regression model to assess the adjusted odds of mortality at a given level as compared to events with no deaths. Analyses were performed using Stata Statistical Software, Version 11.0 11

***Systematic Literature Review***

Key word searches in MEDLINE (Ovid Technologies, humans), EMBASE (Elsevier, B.V., humans), SCOPUS (Elsevier B.V., humans), and Web of Knowledge, Web of Science (Thomson Reuters) were performed to identify articles published in July 2007 or earlier that described natural hazards and their impact on human populations. Following the systematic review, a hand search was conducted to identify relevant articles published after the initial search thru October 2012. One search was done for all the five natural hazards described in this set of papers. This paper describes the results for earthquakes. The systematic review is reported according to the PRISMA guidelines. The key word search included *natural hazard(s), natural disaster(s), volcano(s), volcanic, volcanic eruption, seismic event, earthquake(s), cyclone(s), typhoon(s), hurricane(s), tropical storm(s), flood(s), flooding, mudslide(s), tsunami(s), and tidal wave(s)*. Key words included for impact on human populations were *affected, damage(d), injury, injuries, injured, displaced, displacement, refugees, homeless, wounded, wound(s), death(s), mortality, casualty, casualties, killed, died, fatality, fatalities* in either the title, abstract or as a subject heading/key word. The search resulted in 2,747 articles from MEDLINE, 3,763 articles from EMBASE, 5,219 articles from SCOPUS, and 2,285 articles from ISI Web of Knowledge. Results from the four databases were combined and duplicates excluded to yield a total of 9,958 articles.

Title screening was performed to identify articles that were unrelated to natural disasters or human populations. Each title was screened by two reviewers and was retained if either or both reviewers established that inclusion criteria were met. Percent agreement was assessed across reviewers, and title screening began after 80% agreement was achieved. A total of 4,873 articles were retained for abstract review. During abstract screening articles that met one or more of the following criteria were excluded: language other than English; editorial or opinion letter without research-based findings; related to environmental vulnerability or hazard impact but not human populations; individual case report/study; focus on impact/perceptions of responders; and not related to human or environmental vulnerabilities or impacts of hazards. Each abstract was screened by two reviewers and was retained if either or both reviewers established that inclusion criteria were met. Again, 80% agreement between reviewers was achieved prior to screening. During the abstract review, included abstracts were coded for event type, timeframe, region, subject of focus, and vulnerable population focus. A total of 3,687 articles were retained for full article review. Articles discussing the impacts of natural disasters on human populations in terms of mortality, injury, and displacement were prioritized for review. From this general review of 395 articles specific to earthquake events meeting the aforementioned subject focus criteria were identified for full review. Upon full review, 150 articles were retained 143 that underwent standard data abstraction; seven that were identified as review articles (Figure 1). Articles that focused on risk factors for specific types of injuries (primarily crush injuries and renal failure) or deaths were excluded because they did not provide insight on overall risk factors for mortality or injury. In total, 70 articles relating to risk factors for mortality, injury or displacement were identified; summaries of articles with primary data (n=60) and review articles (n=10) are presented in Tables 1 and 2, respectively.

**Overview of the systematic literature review process for earthquakes:**
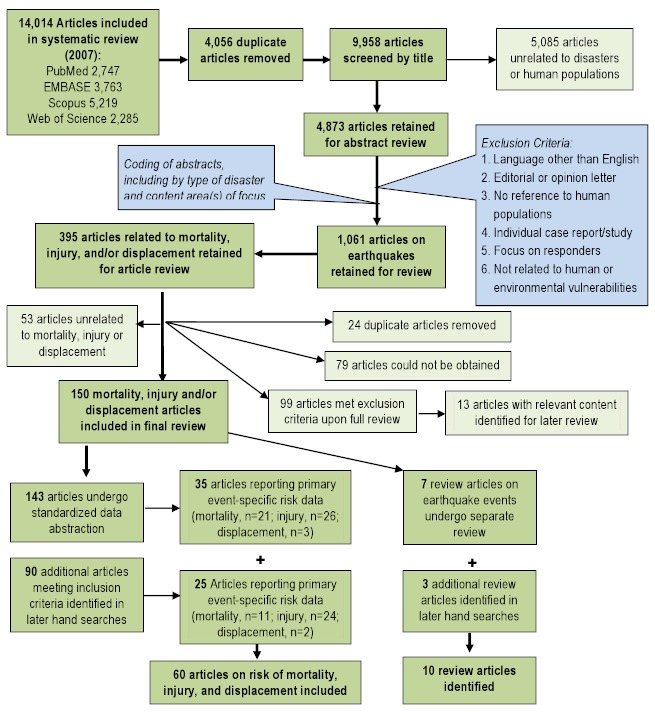

**Table 1: Articles included in the earthquake systematic literature review related to mortality and injury risk (N=60)*:** * Displacement is excluded from the table because primary data on displacement in earthquake events was collected in only six studies: Daley, 2001; Parasuraman, 1995; Roces, 2002, Chun 2010; Kun 2010; and Milch, 2010. ** Additional articles identified in the hand search conducted through October, 2012.

**Publication**	**Event(s)**	**Magnitude**	**Study Summary**	**Mortality**	**Injury**
Armenian, 1992^13^	Dec 7 1988, Armenia	6.9	Case-control study of injuries from the 1988 earthquake in Armenia	NR	x
Pointer, 1992^14^	Oct 17 1989, Loma Prieta, California	7.1	Retrospective review of medical records after the Loma Prieta earthquake	x	x
Roces, 1992^15^	July 16 1990, Philippines	7.7	Unmatched case-control study of those injured/dead from earthquake vs. those uninjured in same neighborhood	x	x
Bissell, 1994^16^	Apr 22 1991,Limon, Costa Rica	7.4	Assessment of medical aspects of the disaster response following the 1991 Costa Rica Earthquake	x	x
Pretto et al., 1994^17^	Apr 22 1991,Limon, Costa Rica	7.4	Retrospective structured interview to investigate risk injury factors and causes and circumstances of prehospital death after the Costa Rica earthquake in 1991	x	NR
Eberhart-Phillips, 1994^18^	Oct 17 1989, Loma Prieta, California	7.1	Medical record review for all investigated deaths from 7 CA counties for 15 days after the Loma Prieta Earthquake	x	NR
Parasuraman, 1995^19^	Sep 30 1993, Maharashtra India	6.4	Quantitative survey assessment of the loss of life and damage to property from the Latur-Osmanabad earthquake in India	x	x
Tanida, 1996^20^	Jan 17 1995,Kobe, Japan	7.2	Analysis of mortality from the Hanshin-Awaji earthquake focused on the elderly	x	NR
Teeter, 1996^21^	Jan 17 1994, Northridge California	6.8	Evaluation of illnesses and injuries in the aftermath of the Northridge earthquake	NR	x
Angus, 1997 22	Mar 13 1992, Ercinzan, Turkey	6.8	Retrospective medical record review of mortality and its relationship to building collapse patterns and initial medical response following the earthquake	x	x
Armenian, 1997^23^	Dec 7 1988, Armenia	6.9	Cohort study of injuries and deaths caused by the Armenian earthquake	x	x
Kloner, 1997^24^	Jan 17 1994, Northridge, California	6.8	Population-based analysis of the effect of the Northridge Earthquake on Cardiac Death in Los Angeles County, California	x	NR
Kuwagata,1997^25^	Jan 17 1995,Kobe, Japan	7.2	Medical record review of final outcome of patients who suffered trauma in the Hanshin-Awaji earthquake	x	x
Peek-Asa, 1998^26^	Jan 17 1994, Northridge California	6.8	Description of fatalities and hospitalized injuries and risk factors from the Northridge earthquake	x	x
Salinas, 1998^27^	Jan 17 1994, Northridge, California	6.8	Comparison of emergency department admissions before and after the Northridge earthquake	NR	x
Shoaf, 1998^28^	1987, 1989 and 1994 California	5.9, 7.1, 6.8	Household survey of Californians residents about three earthquakes, and analysis of injuries and socio-demographic predictors	NR	x
Tanaka, 1999^29^	Jan 17 1995,Kobe, Japan	7.2	Overview of the morbidity and mortality of hospitalized patients during the 15-day period following the Hanshin-Awaji earthquake	x	x
McArthur, 2000^30^	Jan 17 1994, Northridge California	6.8	Evaluation of the burden of injuries resulting in hospitalization in the Northridge Earthquake and the disruption of the usual pattern of service requirements	NR	x
Peek-Asa, 2000^31^	Jan 17 1994, Northridge California	6.8	Analysis of earthquake-related and geographic data with the spatial and geographical relationships resulting from fatal and hospitalized injuries during the earthquake	x	x
Iskit, 2001^32^	Aug 17 1999, Marmara Turkey	7.6	Retrospective analysis of clinical and laboratory data of pediatric trauma patients referred to a tertiary center after the 1999 Marmara earthquake	NR	x
Liang, 2001^33^	Sep 21 1999, Taiwan	7.3	Analysis of risk factors for morbidity and mortality caused by the 1999 Taiwan earthquake	x	x
Mahue-Giangreco, 2001^34^	Jan 17 1994, Northridge California	6.8	Evaluation of the associations between potential risk factors for earthquake-related injuries and injury severity from emergency department data from the Northridge earthquake	NR	x
Roy, 2002^35^	Jan 26 2001, India	7.7	Survey of victims in a hospital assessing injury and other impacts due to earthquake	NR	x
Chan, 2003^36^	Sep 21 1999, Taiwan	7.3	Investigation of earthquake mortality patterns and post-earthquake mortality changes	x	NR
Jain, 2003^37^	Jan 26 2001,Gujarat, India	7.7	Description of evolution of presenting injuries in types of pediatric surgery required; propose an effective disaster relief team composition and strategy	NR	x
Liao, 2003^38^	Sep 21 1999,Taiwan	7.3	Examination of the association between ground motion and structural destruction that causes fatal injuries from the Taiwan quake.	X	NR
Peek-Asa, 2003^39^	Jan 17 1994 Northridge, California	6.8	Population based case-control study to examine how individual characteristics, building characteristics, and seismic features of the earthquake contribute to physical injury.	NR	x
Aoki, 2004^40^	Jan 17 1995,Kobe, Japan	7.2	Assessment of death patterns, cause, and preventability and estimates costs of enhancing the emergency medical services response to prevent unnecessary deaths	x	NR
Chou, 2004^41^	Sep 21 1999, Taiwan	7.3	Examination of risk factors for mortality from the 1999 Taiwan earthquake	x	NR
Ellidokuz, 2005^42^	Feb 3 2002, Turkey	6.1	Cross-sectional study of survivors focusing on risk factors for deaths and non-fatal injuries	x	x
Emami., 2005^43^	Dec 26 2003,Bam, Iran	6.6	Discussion of strategies used to manage a large number of casualties entering one hospital in a short period of time, both in an earthquake or other situation	NR	x
Pawar, 2005^44^	Jan 26 2001, India	7.7	Examination of casualty rates after the earthquake in the Bhuj block.	X	NR
Uzun, 2005 45	Aug 17 1999, Marmara, Turkey	7.6	Investigation of clinical, demographic, and electromyographic characteristics of 12 pediatric quake victims and compare findings with adults.	NR	x
Hatamizadeh, 2006^46^	Dec 26 2003,Bam, Iran	6.6	Review of Bam earthquake epidemiology from a nephrologic perspective; compares complications and outcomes of victims with and without renal failure	x	x
Sabzehchian, 2006^47^	Dec 26, 2003 Bam, Iran	6.6	Analysis of pediatric trauma at tertiary-level hospitals following the earthquake	NR	x
Dhar et al., 2007^48^**	Oct 8 2005, Pakistan	7.6	Medical record review of injuries and deaths of 468 patients admitted to a hospital following the Pakistan earthquake	x	x
Laverick, 2007^49^**	Oct 8 2005, Pakistan	7.6	Analysis of injuries and deaths among 2721 adults and 1449 children in a hospital after the Pakistan earthquake	x	x
Ganjouei, 2008^50^**	Dec 26 2003,Bam, Iran	6.8	Retrospective review of medical records of 1250 injured hospital patients seen after the earthquake	NR	x
Mohebbi., 2008^51^**	Dec 26, 2003Bam, Iran	6.6	Assessment of demographic characteristics, injury, treatment and outcomes of 854 earthquake victims	NR	x
Mulvey, 2008^52^**	Oct 8 2005, Pakistan	7.6	Retrospective review of medical records to document injury patterns in the first 72 hours after the Kashmir earthquake	x	x
Bai, 2009^53^**	Oct 8 2005, Pakistan	7.8	Retrospective analysis of injuries of 2194 patients from the Pakistan earthquake	NR	x
Doocy, 2009^54^**	Aug 15 2007, Peru	8.0	Population-based cluster survey of households affected by earthquake to assess earthquake-related risk and vulnerability	x	x
Najafi , 2009^55^**	Dec 26 2003,Bam, Iran	6.6	Retrospective analysis of demographic characteristics, biochemical markers and outcomes of individuals referred for medical care after the Bam earthquake	NR	x
Sami, 2009^56^**	Oct 8 2005, Pakistan	7.6	Random sample of 310 hospital patients to assess demographics and injury types	NR	x
Wen, 2009^57^**	May 12 2008, China	7.9	Hospital-based case-control study of deaths due to earthquake injuries to assess the determinants of earthquake-related mortality	x	x
Xiang, 2009^58^**	May 12 2008, China	7.9	Medical record analysis of pediatric victims’ characteristics, injury type, and resuscitation	NR	x
Yang, 2009^59^**	May 12 2008, China	7.9	Retrospective medical record review of injured patients following the China earthquake	NR	x
Yasin, 2009^60^**	Oct 8 2005, Pakistan	7.6	Medical record review of injuries, deaths, complications and procedures	x	x
Zhang, 2009^61^**	May 12 2008, China	7.9	Retrospective record review of demographics and injury from 1170 patients following the China earthquake	NR	x
CDC, 2010^62^**	Jan 12 2010, Haiti	7.0	Medical record review of injuries and patient characteristics at a field hospital in Haiti	NR	x
Jian, 2010^63^**	May 12 2008, Wenchuan China	7.9	Retrospective record review of demographic characteristics and injuries of 196 hospital patients	NR	x
Milch, 2010^64^**	Aug 15 2007, Peru	8.0	Household survey and observational damage assessment to evaluate associations between social and environmental determinants of injury and displacement	NR	x
Qiu, 2010^65^**	May 12 2008, China	7.9	Medical record review of injury cause, type and treatment and patient demographic characteristics from 11 hospitals	NR	x
Sullivan, 2010^66^**	Oct 8 2005, Pakistan	7.6	Cross-sectional surveys to assess risk factors for earthquake related mortality	x	NR
Farfel, 2011^67^**	Jan 12 2010, Haiti	7.0	Analysis of injuries sustained by pediatric patients in a field hospital	NR	X
Zhao, 2011^68^**	May 12 2008, China	8.0	Review of children treated by the relief team.	X	X
Ardagh, 2012^69^**	Feb 22 2011, New Zealand	6.3	Data from Christchurch hospital extracted from an electronic database for review.	NR	X
Kang, 2012^70^**	April 14 2010, China	7.1	Medical records of 3,255 patients from 57 hospitals were analyzed retrospectively.	NR	X
Sudaryo, 2012^71^**	Sept 30 2009, Indonesia	7.6	Prospective cohort study of inured patients over a 6 month period in Padang, Indonesia.	X	X
Tan, 2012^72^**	Sept 30, 2009,Indonesia	7.6	Two Singapore Armed Forces (SAF) primary healthcare clinics prospectively collected patient medical information for comparison..	X	X

**Table 2: Review articles relating to earthquake mortality and injury (N=10):** * Additional articles identified in the hand search conducted through October 2012

**Article**	**Summary**	**Key Findings**
White & Harlow, 1993^73^	Catalog of human impacts from 51 earthquakes in Central America from 1900-1991	Upper-crust earthquakes (n=51) caused at least 40,500 deaths, 105,000 injuries and made 900,000 homeless in Central America from 1900-1991. Of earthquakes with magnitude >6, 23 of 30 occurred along the Central American volcanic front. Destructive upper-crust earthquakes occurred on average every 2.5 years. Subduction zone earthquakes can have larger magnitudes and produce more widespread damage, but volcanic-front earthquakes are more frequent and pose greater risk because they occur closer to densely populated areas.
Alexander, 1996^74^	Review of 83 earthquakes from 1993-1996	Deaths and injuries occurred in at least 40 and 42 earthquakes, respectively. Most deaths and injuries (86% and 97%, respectively) were caused by earthquakes with 6.5-7.4 magnitude and occurred between midnight and 6 AM (94% and 77%, respectively). Building collapse was the primary cause of death and injury; in 23 earthquakes, running out of doors in panic was mentioned, which can increase risk of injuries and deaths.
Musson, 2003^75^	Review of fatal earthquakes in Britain from 974-2003	Of the ten fatal earthquakes that occurred in Britain from 974 to 2003, only 10 were directly attributable to the earthquake event. Six were due to falling stones/rock and four due to building damage. There was no correlation between magnitude and mortality.
Bird & Bommer, 2004^76^*	Summarizes social and economic losses in 50 earthquakes,1989-2004	Compared to fault rupture, tsunami, liquefaction, and landslide, ground shaking is the principal cause of damage and loss in earthquakes. Land use, land zoning, improper construction on liquefiable soil, and design and construction are risk factors for injury and death.
Srivastava & Gupta, 2004^77^	Review of timing, after-shocks and magnitude of 503 earthquakes >5.5 magnitude in India from 1800-2001	Earthquake timing and aftershocks are important factors related to earthquake mortality. Earthquakes that occur during the night or early morning cause more deaths than earthquakes that occur during the day. In evening/night earthquakes, mitigation efforts are hampered by decreased visibility, falling debris and electricity outages. Some regions of India are more prone to severe earthquakes than others due to geological location.
Fu et al., 2005^78^	Review of characteristics of 420 shallow, strong earthquakes that were associated with fatalities in China from 1901-2001	From 1901 to 2001 the majority of earthquakes that caused harm to humans in China were shallow and strong; these earthquakes (n=420) caused at least 604,677 deaths. Most earthquakes with magnitude >6 occurred in Western China; the two deadliest were in 1920 (246,269 deaths; magnitude 8.6; Haiyuan, Ningxia) and 1976 (250,723 deaths; magnitude 7.8; Tangshan, Hebei). The main cause of death was building collapse; risk factors for death included time of day, building damage levels and population density. A non-linear relationship between magnitude and mortality was also observed.
Gutierrez et al., 2005^79^	Multivariate analysis of mortality using demographic, seismic and geographic parameters in 366 earthquakes, 1980-2001	Between 1980 and 2001, 553,000 injuries and 190,000 deaths were reported in 366 earthquakes. A multivariate mortality prediction method was proposed that includes physical and geographic location, human population, GDP per capita, and magnitude. As magnitude increased mortality increased; and as depth increased, mortality decreased. However, high magnitude may not induce high mortality if it is not combined with key physical and demographic criteria. Rural and semi-rural areas with poorly built environments had higher mortality.
Spence, 2007^80^	Review of earthquakes from 1960-2006 focusing on earthquake mortality and affected countries’ earthquake risk mitigation and prevention strategies	Between 1960 and 2006, the ten most lethal earthquakes caused 80% of the 1 million earthquake deaths and occurred in low- and middle-income countries. The main cause of death was building collapse; unreinforced masonry buildings were associated with higher death tolls. Efforts to control and reduce earthquake mortality have made progress in wealthier earthquake prone countries but little or no progress in low- and middle-income countries. Recent experience of a strong earthquake and availability of resources for mitigation were the two strongest determinants of action for risk mitigation. Growing urbanization and populations in developing countries have increased the risk of human impacts. Establishing and implementing building standards is the most important strategy for mortality and injury reduction.
Gautschi et al., 2008^81^*	Review of individual and population impacts of major earthquakes from the 20^th^ and 21^st^ centuries and mitigation strategies	Reviews earthquakes with the most deaths and injuries from 20^th^ and 21^st^ century and describes common earthquake injuries and effective treatment approaches. In recent earthquakes mortality was significantly higher in intensive care patients treated in local earthquake-affected hospitals then those treated in unaffected hospitals. In order to minimize trauma-related mortality, knowledge of local medical facilities, equipment, capacity, and transportation infrastructure are important as is a medical transport corridor.
Chan et al., 2010^82^*	Review of the human impact of earthquakes in China from 1906-2007	China has had the greatest human impact from earthquakes of any country in the past century. This review summarizes the mortality tolls from earthquakes in China and other major earthquakes from 1906 to 2007 and identifies gaps in the literature including lack of research on mortality and morbidity risk factors and populations with chronic disease.

## Results

***Historical Event Review***

Overall, 74.1% of events in the database were reported by EM-DAT, 60.8% by NOAA and 25.8% by USGS; reporting by USGS improved dramatically from 2000 onwards. An average of 24.6 (range 13-43) earthquakes affected human populations annually between 1980 and 2009 (figure 2). The frequency of events increased over time, which is attributable to improvements in reporting. The average magnitude was 6.2 (range 4-9; n=493, 66.8% reporting) and focal depth 27.1km (median 19.0, range 0-235.8; n=493; 66.8% reporting). Earthquake mortality increased in parallel with the frequency of events from the 1980s onwards (Figure 3). A rapid increase in earthquake-affected populations was observed after 2000, which is likely a result of both improved reporting and population growth (Figure 3). Earthquakes were most frequent in the Americas, South-East Asia and the Eastern Mediterranean with each region accounting for 20-25% of events; however earthquake impact was greatest in the Western Pacific, which accounted for 44% of deaths and Americas, which accounted for 60% of the affected population (Figure 4). The overall impact of earthquakes on human populations is summarized in Table 3. Of the 738 identified events, the databases recorded 687 (96.9%) causing deaths, 420 (56.9%) causing injuries and 359 (51.4%) leading to homelessness.

**Reporting of earthquakes by source and decade (n=953):**
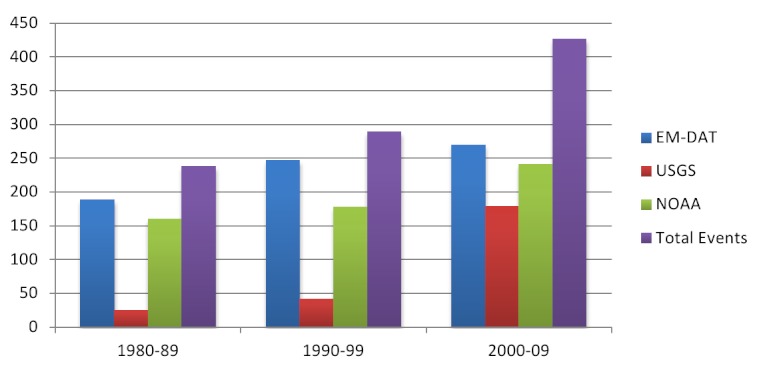


**Earthquakes affecting human populations by decade, 1980-2009 (n=738):**
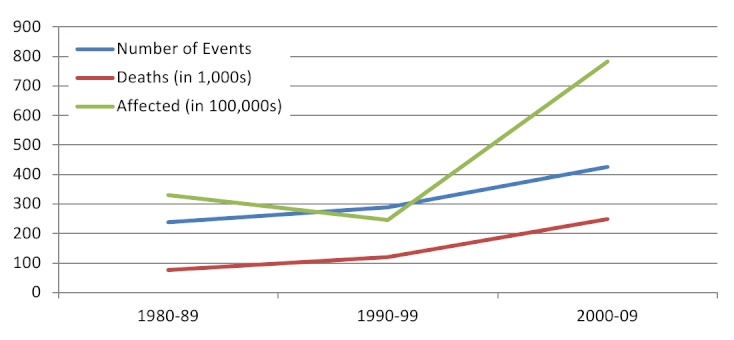

**Earthquakes and their impact on human populations by region, 1960-2009*:**
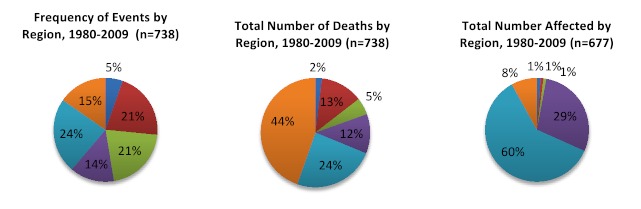
*Regions as defined by the World Health Organization

**Table 3: Summary measures for the impact of earthquakes on human populations, 1980-2009 (n=738):** Notes: Best estimate figures are based on the average reported number of deaths or injuries in an event; homeless and affected populations were rarely reported by sources other than EM-DAT thus ranges are not presented.

***Reported Overall Impact of Earthquakes***							
***Human Consequence***	**# of Events**			**Best Estimate**		**Range**	
Deaths	687			372,634		314,531-412,599	
Injuries	417			995,219		845,345-1,145,093	
Homeless	376			16,003,542		---	
Affected	688			61,521,492		---	
***Event Summary Statistics***							
***Human Consequence***		**# of Events**		**Median**	**Mean **		**Range**
***Deaths (all sources)***		**687 (93.1%)**					
Reported by EM-DAT		686	93.0%	2	554		0-87,476
Reported by NOAA		366	49.6%	9	933		1-87,652
Reported by USGS		127	17.2%	0	1,289		0-87,350
***Injuries (all sources)***							
Reported by EM-DAT		324	43.9%	100	2,593		1-166,812
Reported by NOAA		296	40.1%	60	21,614		1-374,171
Reported by USGS		69	9.4%	100	19,641		0-166,836
***Homeless (all sources)***							
Reported by EM-DAT		372	50.4%	0	297,140		0-5,000,000
Reported by		16	2.2%	46,594	970,495		328-4,000,000
***Affected Population*** * (EM-DAT)*		**677**	**91.7%**	**3152**	**200,783**		**1-46,000,000**

*Affected Population and Homelessness.* An estimated 61.5 million people were affected by earthquakes between 1980 and 2009, including 16 million rendered homeless. These figures are likely to underestimate the true impact of earthquakes because the total affected and homeless populations were reported in only 95.5% and 51.4% of events, respectively. There were an average of 200,783 affected (median 3,7215, range 1-46,000,000) and 46,594 homeless (median 0, range 0-4,000,000) per event [where data were reported] and distributions were highly skewed.

*Mortality and Injury.*When mortality data from the three sources were combined, information on deaths (inclusive of events with no reported deaths) was recorded in 93.1% (n=687) of earthquakes, with fatalities occurring in 71.0% (n=524). Overall, an estimated 372,634 total deaths (range: 314,531-412,599) were reported; for events with mortality, there were an average of 604 deaths (median=3, range=0-87,652) per event when using the highest reported death toll. Deaths were concentrated in the Western Pacific (190,955 deaths, 44.6%) and Americas (103,679, 24.2%). When assessed by country, the greatest earthquake mortality was observed in China (n=90,106, 21.0%), Pakistan (n=88,314, 20.6%), and Iran (n=86,254, 20.1%). The two most deadly earthquakes (Sichuan China, 2008 and Kashmir Pakistan, 2005) accounted for 40% of all earthquake mortality.

Injuries were reported in only 56.9% (n=420) of events with estimated total of 995,219 reported injuries (range: 845,345-1,145,093). When injuries were reported, there were an average of 3,499 (median=100, range 1-374,171) injuries per event using the highest reported number for the event. To better estimate the total number of injuries, it was presumed that injuries would occur in events where deaths were reported. Among the 687 earthquakes with fatalities reported, injuries were reported in only 420 (61%) events. When the median and mean for injuries were applied to the remaining 267 events, between 29,392 and 1,267,864 additional unreported earthquake related injuries may have occurred between 1980 and 2009.

Bivariate associations between country-level characteristics and earthquake-related mortality are presented in Table 4. All predictors except for earthquake focal depth were significantly associated with mortality. In the adjusted multinomial logistic regression model (Table 5), only magnitude was significantly associated with earthquake mortality. World Bank development level, the Gini Index coefficient, and focal depth were not statistically associated with earthquake-related mortality. The odds of a high mortality event as compared to an event with no deaths increased by 11.93 (95 CI: 5.35-26.57) per additional point on the magnitude scale.

**Table 4. Earthquake Mortality by Select Country Characteristics, 1980-2009 (N=738):** *GINI coefficient scores for income distribution range from 0 to 100 with 0 representing a perfect equality and 100 perfect inequality.

**Characteristic**	**No deaths (*n=* 214)**	**1-9 deaths (*n* =258)**	**10-99 deaths (*n* =144)**	**>** **100 deaths (*n* =122)**	**p-value**
**Decade**, n (%)							
1980-89	50 (23.3%)	69 (26.7%)	42 (29.2%)	33 (27.1%)	0.008
1990-99	71 (33.2%)	82 (32.8%)	57 (39.6%)	58 (47.5%)	
2000-09	93 (43.5%)	107 (41.5%)	45 (31.3%)	31 (25.4%)	
** World Health Organization Region**, n (%)							
Europe	64 (29.9%)	49 (19.0%)	27 (18.8%)	16 (13.1%)	0.001
Americas	38 (17.8%)	65 (25.2%)	30 (20.8%)	21 (17.2%)	
Africa	6 (2.8%)	18 (7.0%)	9 (6.3%)	7 (5.7%)	
South-East Asia	25 (11.7%)	31 (12.0%)	19 (13.2%)	27 (22.1%)	
Western Pacific	54 (25.2%)	63 (24.4%)	32 (22.2%)	24 (19.7%)	
Eastern Mediterranean	27 (12.6%)	32 (12.4%)	27 (18.8%)	27 (22.1%)	
**World Bank Development Level**, n (%)							
Low-income	14 (6.5%)	26 (10.1%)	14 (9.7%)	18 (14.8%)	0.026
Lower-middle income	104 (48.6%)	121 (46.9%)	73 (50.7%)	67 (54.9%)	
Upper-middle income	47 (22.0%)	69 (26.7%)	38 (26.4%)	26 (21.3%)	
High-income	49 (22.9%)	42 (16.3%)	19 (13.2%)	11 (9.0%)	
GINI*(n=681)							
Mean (SD)	39.3 (6.4)	41.2 (8.0)	41.3 (7.6)	41.0 (7.0)	0.0241
** Per Capita GDP **(US$) (n=732)							
Mean (SD)	11,777.1(14,911.8)	8975.9 (12,854.8)	7387.1 (10969.7)	6,058 (10,487.3)	0.0013
** Focal Depth**, km (n=493)							
Mean (SD)	22.9 (21.1)	30.2 (33.3)	25.5 (25.6)	27.9 (34.1)	0.2228
** Magnitude **(n=493)							
Mean (SD)	5.9 (0.7)	6.3 (0.6)	6.2 (0.8)	6.7 (0.7)	<0.001

**Table 5: Multinomial Logistic Regression: Relative Risk Ratios (RRR) of earthquake mortality* (N=288):** *Reference is ‘no deaths’ for all categories (n=55) **Model includes both magnitude and focal depth; focal depth is measured on a log (base 10) scale ***p-values reported for each category with Wald test p-values for the variable.

**Characteristic**	**Low Mortality 1-9 deaths (*n* =127)**		**Medium Mortality 10-99 deaths (*n* =89)**		**High Mortality 100 deaths (*n* =72)**	
	RRR (95 CI)	p-value	RRR (95 CI)	p-value	RRR (95 CI)	p-value***
**Decade**						
1980-89	*Reference*		*Reference*		*Reference*	0.027
1990-99	1.03 (0.42, 2.56)	0.947	.98 (.36, 2.42)	0.907	0.52 (0.19, 1.44)	0.21
2000-09	0.98 (.42,2.32)	0.97	0.46 (0.18, 1.16)	0.10	0.19 (0.06, 0.55)	**0.002**
**WHO Region**						<0.001
Africa	*Reference*		*Reference*		*Reference*	
Europe	<0.001 (0,0)	<0.001	<0.001 (0,0)	<0.001	<0.001 (0,0)	<0.001
Americas	<0.001 (0,0)	<0.001	<0.001 (0,0)	<0.001	<0.001 (0,0)	<0.001
South-East Asia	<0.001 (0,0)	<0.001	<0.001 (0,0)	<0.001	<0.001 (0,0)	<0.001
Western Pacific	<0.001 (0,0)	<0.001	<0.001 (0,0)	<0.001	<0.001 (0,0)	<0.001
E Mediterranean	<0.001 (0,0)	<0.001	<0.001 (0,0)	<0.001	<0.001 (0,0)	<0.001
**Gini Coefficient**	1.10 (0.99, 1.20)	**0.054**	1.04 (0.95, 1.15)	0.156	1.11 (0.99, 1.24)	0.058
**Per Capita GDP (1000s)**	1.01 (.97, 1.05)	0.646	.98 (.93, 1.02)	0.302	1.00 (.95, 1.05)	0.97
Focal Depth km)**	1.82 (.57,5.9)	0.314	.58 (.16, 2.05)	0.394	.38 (0.09, 1.58)	0.184
**Magnitude**	2.8 (1.39, 5.62)	**0.004**	3.99 (1.9, 8.3)	**<0.001**	11.93 (5.35, 26.57)	**<0.001**

***Systematic Literature Review***

*Mortality.* Of the articles reviewed, 27 reported on causes of death (n=17) and/or risk factors including sex (n=8), age (n=15), building or location (n=17) and other risk factors (n=11) (Table 6). The primary cause of death in the majority of studies was building collapse 16 ^-^ 20 ^,^ 22 ^,^ 23 ^,^ 25 ^,^ 29 ^,^ 31 ^,^ 33 ^,^ 36 ^,^ 40 ^,^ 46. Females faced a significantly increased risk of death in three studies 19 ^,^ 36 ^,^ 41 while several others found no significant difference in mortality by sex 18 ^,^ 33 ^,^ 38 ^,^ 66 none reported that males faced a significantly increased mortality. Of the 14,709 earthquake deaths where sex was reported in the articles reviewed, 45% (n=6649) and 55% (n=8,060) of deaths were among males, yielding a ratio of 1.0:1.2 for male to female mortality. Age was a risk factor for mortality in many studies. Older populations consistently had higher rates of death 13 ^,^ 18 ^,^ 20 ^,^ 26 ^,^ 29 ^,^ 33 ^,^ 36 ^,^ 38 ^,^ 41 ^,^ 66 and in several studies children also faced increased mortality 19 ^,^ 29 ^,^ 33 ^,^ 44.

**Table 6: Articles Reporting Detailed Information on Mortality (n=29):** 

**Article**	**Event**	**Mortality Rate**	**Cause**	Sex	**Age**	**Building and Other Risk Factors**
Angus, 1997^22^	Turkey, 1992	Not reported	48% (n=26) instant/building collapse. Of protracted deaths, 50% (n=13) hemorrhaged and 42% (n=11) asphyxiated.	Not reported	Not reported	92% of indoor deaths occurred in mid-level unreinforced masonry buildings; deaths were more likely among those on the ground floor. Prior first-aid or rescue training of lay, uninjured survivors was associated with a higher likelihood of rescue and resuscitation.
Aoki, 2004^40^	Japan, 1995	Not reported	Asphyxia/pressure, 74% (n=3551); contusion injury, 17% (n= 828); head/neck injury, 8% (n=286); indirect, 3% (n=121).	Not reported	Not reported	Not reported
Armenian, 1997^13^	Armenia, 1988	254/10,000	Trauma due to building collapse.	Not reported	Increased deaths among >60yrs.	Building height and upper floor location were important predictors of death; odds of death were 9.8 times greater for those inside compared witho those outside.
Bisselll, 1994^16^	Costa Rica, 1991	Not reported	Entrapment and crush injury.	Not reported	Not reported	Most fatalities occurred in homes; wood-frame houses with lateral bracing were less likely to collapse than those without bracing.
Chan, 2003^36^	Taiwan, 1999	134/10,000	77% (n=1,441) instant/building collapse; causes asphyxiation (32%), intracranial inj (29%), trauma/ trunk/extremity inj (16%), internal inj (7%), crush inj (6%), and fractures (6%).	Male: 122/10,000; Female: 144/10,000. Ratio: 0.85:1.0	Increased risk w/ age; death ratio of >45yrs to adults <45yrs of 3.3:1.	Complete collapse was a better predictor of death than partial collapse; a 1% increase in completely collapsed home was associated with a 5% increase in the crude death rate.
Chou, 2004^41^	Taiwan, 1999	Not reported	Not reported	Increased female risk: OR 1.2 (CI: 1.1-1.3)	Higher mortality in older age groups.	People with lower socioeconomic status and the physically disabled were at increased risk of mortality.
Doocy, 2009^54^	Peru, 2007	1.4/1000	Not reported	Not reported	Sample size too small for analysis	Not reported
Eberhart-Phillips,1994^18^	California, 1989	Not reported	Elevated freeway collapse; 81% (46/57) of direct deaths vehicle /roadway related. Indirect deaths due to CO poisoning, heart attacks, falls and GI bleeding.	Victims more likely to be female (NS)	Victims more likely to be older (NS)	Elevated freeway collapse
Ellidokuz, 2005^42^	Turkey, 2002	1.6/10,000	Not reported	Not reported	Median age = 51 yrs (range 4-74)	Collapsed (11 deaths) and severely damaged (2 deaths) buildings.
Hatamizadeh, 2006^46^	Iran, 2003	19.7% (41/2086 patients)	Trauma (n=11), cardiac arrest (n=6), septicemia (n=5), DIC (n=3), hypovolemic shock (n=2), and ARDS (n=3)	Not reported	Mean age = 32.3 yrs (SD=16.3)	Patients with acute renal failure were significantly more likely to die than those with other diagnoses.
Kuwagata, 1997^25^	Japan, 1995	6.6%(178 /2702 injuries)	Crush syndrome (n=50, 28%), vital organ injuries (n=36, 20%), 7 (1%); fractures of the pelvis or spine (n=7, 1%); others (n=18, 1.3%); and unknown (n=67, 59%)	Not reported	Not reported	The most life-threatening injuries, crush syndrome and vital organ injuries, occurred indoors.
Laverick, 2007^49^	Pakistan, 2007	Adults: 4.3% (118/ 2721) Children 3.5% (50/ 1449 patients)	Tetanus (n=22) deaths and neonatal causes (n=4); no other causes reported	Not reported	Not reported	Not reported
Liang, 2001^33^	Taiwan, 1999	14.82/100,000	Body compression, including head injury (32%), shock (29%) and asphyxiation (29%). Other causes included organ injury, spinal cord injury, burns and CO poisoning.	Male:14.0/100,000 Female:15.6/100,000 Ratio:1.11 (NS)	>80yrs 80/100,00070-79yrs 50/100,000 20-29yrs 6.9/100,000 0-9yrs 12.7/100,000.	Distance to epicenter; earthquake intensity, age, population density, and physicians and hospital beds per 10,000 population were all significant predictors of mortality.
Liao, 2003^38^	Taiwan, 1999	Not reported	Not reported	Male:10.7/100,000 Female:10.1/100,000 Ratio: 1.05(NS)	<15yr:8.5/100,00015-64:8.4/100,000 65+: 34.8/100,000	Overall building collapse rate was a better predictor of mortality than partial building collapse. Intensity and distance to the epicenter were positively associated with mortality.
Mulvey, 2008^52^	Pakistan, 2008	0.2% (2/1502 patients)	Head injuries	Not reported	Not reported	Not reported
Parasuraman, 1995^19^	India, 1993	Not reported	Building collapse	More females than males died in all adult age groups.	By age grp: <14, 50%; 15-24, 13%; 25-59, 28%; 60+, 11%.	Homes with mud/stone walls suffered the most damage (~90% collapsed); fewer deaths occurred in mud/thatch homes and in stone/mud/concrete homes.
Pawar, 2005^44^	India, 2001	Not reported	Not reported	Not reported	By age group: 0-14, n=171 (62%); 14-19, n=41 (15%), adults, n=45 (16%); older adults, n=19 (7%).	The death rate was significantly associated with distance to epicenter.
Peek-Asa, 1998^26^	California, 1994	19.3% (33/ 171 injured)	Asphyxia and body compression from building collapse (n=22, 71%); vehicle accidents (n=5, 15%); falls (n=4, 12%).	Not reported	31% of the deceased were >65 yrs.	Most fatalities were caused by a structural failure (n=25, 76%); >66% of fatalities involved a structural failure of the home. Earthquake related motor vehicle injuries were 5 times more likely to result in fatality than a hospitalized injury.
Peek-Asa, 2000^31^	California, 1994	22.6% (30/ 133 injured patients)	Not reported	Not reported	Not reported	Fatal injuries were concentrated near the epicenter and in areas with higher peak ground acceleration.
Pointer, 1992^14^	California, 1989	1.3/100,000 (CMR)	Not reported	Not reported	Not reported	Not reported
Pretto,1994^17^	Costa Rica, 1991	4/10,000	92% (n=45) instant deaths due building collapse/crush syndrome	Not reported	Not reported	People inside wood frame buildings had a higher risk of injury and death than people in other building types (OR 22.5, p<.001).
Roces, 1992^15^	Philippines, 1990	19% among the injured (68/363)	Not reported	Not reported	Not reported	Cases were more likely to be inside concrete/mixed materials rather buildings as compared to wood (OR 2.6, CI 1.7-4.1) and on a middle or upper floor (OR 3.4, CI:2.2-5.5 and OR 1.9, CI 1.3-2.9, respectively). Chance of survival decreased as time of rescue increased: 84% of the survivors were rescued within the first hour.
Sullivan, 2010^66^	Pakistan, 2005	1.7-5.4% in camps/ communities (708 deaths)	Not reported	Higher Death rate among females (NS)	Children <5 (1.2-10.6% CMR) and adults >50 (3.2-9.9% CMR) had highest risk	Not reported
Tanaka, 1999^29^	Japan, 1995	8.6% (527/ 6107 patients)	Crush injuries (n=50). Indirect causes included respiratory (n=110) and cardiovascular (n=56).	Not reported	Increased with age in patients with prior injuries and illness.	Not reported
Tanida, 1996^20^	Japan, 1995	Not reported	Crush injuries (77%); also penetrating injuries and burns.	Among those >60yrs, female fatalities were 2 times greater than males.	>50% of deaths among those >60; the death rate of 80+yrs was 6 times that of <50yrs.	Not reported
Wen, 2009^57^	China, 2008	NA – case control study	Not reported	Not reported	Not reported	Traumatic brain injury, multiple system organ failure, prior disease, and infection significantly associated w/ increased death risk
Yasin, 2009^60^	Pakistan, 2005	1.9% (17/862 patients)	Tetanus (n=7), trauma/ sepsis (n=5), spinal injury n=(2), crush syndrome (n=2), head injury (n=1)	Not reported	Not reported	Not reported
Zhao, 2011^69^	China, 2008	7 deaths	4 patients with open-head injury, 3 had severe crush injury.	Not reported	Not reported	Not reported
Kang, 2012^70^	China, 2010	0.2% (7/3255)	Four patients died from earthquake-related injuries and three from other illnesses.	Not reported	Not reported	Not reported

A majority of deaths occurred indoors 13 ^,^ 16 ^,^ 19 ^,^ 22 ^,^ 25 ^,^ 29 ^,^ 42 often at home, and the rate of complete building collapse was a good predictor of crude mortality rates 36 ^,^ 38 Construction materials were associated with increased mortality risk, including unreinforced masonry 22, mud and stone walls^19^, concrete 15, panel construction 13 and wood construction 16 ^,^ 17 ^,^ 42; however no clear trend was observed across studies. Location on the ground floor 20 ^,^ 22 and upper floors 15 ^,^ 23 of multistory buildings were associated with increased risk of death. Other mortality risk factors included earthquake intensity and distance to epicenter 31 ^,^ 33 ^,^ 38 ^,^ 44 physical disability 41 prior injury or illness 29, low socioeconomic status 41, and being in a car 31. Response and health systems characteristics associated with mortality risk included time to rescue^15^, per capita availability of physicians and hospital beds 33, and prior first-aid or rescue training of lay, uninjured survivors 22; better availability of rescue and early emergency care could prevent a substantial portion of deaths 17.

*Injury. *Detailed information on injury was reported in 51 articles among which 42 included data on injury type, 30 on gender risk, and 31 on age risk (Table 7). Soft tissue injuries (including lacerations and contusions) and fractures were the most common types of injury reported 15 ^,^ 16 ^,^ 23 ^,^ 25 ^,^ 27 ^,^ 29 ^,^ 43 ^,^ 47 ^,^ 51 ^,^ 52 ^,^ 53 ^,^ 56 ^,^ 58 ^,^ 60 ^,^ 62 ^,^ 67 ^,^ 69 ^,^ 70 ^,^ 71, and the extremities were the most likely areas of the body to be affected 26 ^,^ 46 ^,^ 50 ^,^ 51 ^,^ 59 ^,^ 61. Crush injuries/syndrome were reported as the most common injury in several in-patient studies, and there was a substantial body of literature on this topic (articles focusing on a specific injury type were not abstracted for this review) 16 ^,^ 32 ^,^ 45 ^,^ 55 . The proportion of injured by sex and, when combined, suggests a similar injury risk among males in females- Males accounted for the majority in eleven studies 13 ^,^ 32 ^,^ 43 ^,^ 46 ^,^ 48 ^,^ 50 ^,^ 51 ^,^ 52 ^,^ 53 ^,^ 71 ^,^ 73 and females in sixteen 23 ^,^ 26 ^,^ 28 ^,^ 32 ^,^ 34 ^,^ 35 ^,^ 39 ^,^ 45 ^,^ 47 ^,^ 54 ^,^ 56 ^,^ 58 ^,^ 59 ^,^ 62 ^,^ 63 ^,^ 65 ^,^ 70 ^,^ 72 . However statistically significant differences were observed in a few cases, all of which suggested increased female risk 28 ^,^ 39 ^,^ 45 ^,^ 55 . Many articles reported descriptive age data, however information on age-related injury risk was reported in fewer articles and included decreased risk among children 30 ^,^ 46, and increased risk among young and/or working age adults 50 ^,^ 52 ^,^ 56 ^,^ 62 , the elderly 30, and with increasing age 26 ^,^ 29 ^,^ 34 ^,^ 39 ^,^ 54, Building characteristics and other related risk factors included being indoors 13 ^,^ 15 ^,^ 16 ^,^ 23 ^,^ 25, or on a middle or upper floor 13 ^,^ 15 ^,^ 23 ^,^ 25, construction type and/or quality 15 ^,^ 16 ^,^ 23 ^,^ 26 ^,^ 34 ^,^ 37 ^,^ 39 and low socioeconomic status 54 ^,^ 56.

**Table 7: Articles Reporting Detailed Information on Earthquake-Related Injuries (n=50)*:** Notes: Peek-Asa 2000 and Laverick, 2007 reported detailed information on injury but are excluded from the table because no information was reported on factors included in the table. In many cases reporting by injury type, age, and/or sex was incomplete which is why numbers reported for each outcome may not sum to the total number of deaths reported.

** Article**	**Event**	**Injuries Reported**	**Injuries By Type and Cause**	**By Sex**				**By Age**
				Male		Female		
				N	(%)	N	(%)	
Angus, 1997	Turkey, 1992	29	Not reported	NR	NR	NR	NR	Not reported
Armenian, 1992	Armenia, 1988	189	Not reported	120	63%	69	37%	Descriptive only
Armenian, 1997	Armenia, 1988	1454	Fractures/broken bones (37%) and crush injuries (27%) were most common	658	45%	796	55%	Not reported
Bai, 2009	Pakistan, 2005	2194	Open wounds (68%), soft tissue (20%), and fractures (18%), most often in lower extremity; infection was common.	166	60%	109	40%	Descriptive only
Bissell, 1994	Costa Rica, 1991	182	Crush injuries, long bone fractures and soft tissue injuries were most common	NR	NR	NR	NR	Not reported
CDC, 2010	Haiti, 2010	126	Fractures/dislocations, wound infections, and head, face, and brain injuries were most common.	74	46%	85	53%	Descriptive; young adults were most at risk
Dhar, 2007	Pakistan, 2005	468	Fractures/broken bones (58%), soft tissue only (35%), chest trauma (5%), spine injuries (4%), and others (2%).	271	58%	197	42%	Descriptive only
Doocy, 2009	Peru, 2007	92	Crush injuries (31%), fractures (23%), wounds (20%), other types (18%), and blunt force injury (8%) were most common.		2%		3%	Injury risk increased by 3% per additional year of age.
Ellidokuz, 2005	Turkey, 2002	18	18 injured persons, including 4 with fractures/broken bones and 1 burn patient all had lacerations or contusions.	9	50%	9	50%	Descriptive only
Emami, 2005	Iran, 2003	708	Lacerations/contusions (27%), fractures/broken bones (20%), and crush syndrome (4%) were most common.	392	55%	316	45%	Descriptive only
Farfel, 2011	Haiti, 2010	182	Open wounds (29%), fractures (26%), crush injuries (16%), superficial injuries (16%), contusions (4%), dislocations (3%), and head injuries (3%) were most common.	NR	NR	NR	NR	Descriptive only
Ganjouei, 2008	Iran, 2003	1250	Lower limb (41%), pelvis (26%) and head injuries (25%) were most common among hospitalized patients in the study.	223	54%	193	46%	Risk of injury was highest among 19-60yrs of age and very low among children
Hatamizadeh, 2006	Iran, 2003	2086	Trauma to extremities (36%), head/neck (16%), abdomen (16%), and thorax (9%).	1079	52%	966	46%	Significantly lower injury risk for those <15 yrs (p<.001) and higher risk for young/ middle-aged adults (p<.001).
Iskit, 2001	Marmara Turkey, 1999	33	Crush injuries/syndrome (45%), and fractures/broken bones (24%) were most common.	17	52%	16	48%	Not significant
Jain, 2003	India, 2001	62	Orthopedic injury (42%), soft tissue injury (10%), and burns (6%).	NR	NR	NR	NR	Descriptive only
Jian, 2010	China, 2008	196	Multiple trauma (36%), and lower limb injury (34%) were the most common.	88	45%	108	55%	Descriptive only
Kuwagata, 1997	Japan, 1995	2702	Fractures/broken bones (45%), soft tissue injury (33%), crush syndrome (14%), burns (2%), nerve injuries (2%), other (2%) and unknown (4%).	NR	NR	NR	NR	Not reported
Liang, 2001	Taiwan, 1999	8722	90% suffered from head injury, open wounds, contusions or fractures	NR	NR	NR	NR	Not reported
Mahue-Giangreco, 2001	California, 1994	418	Not reported	167	40%	251	60%	Risk of injury increased with age category (NS); risk of injury was 6 times greater in patients 60+yr compared 30-39yr
McArthur, 2000	California, 1994	138	Not reported	NR	NR	NR	NR	Significantly lower risk among children and higher risk among adults >65yrs
Milch, 2010	Peru, 2007	---	Not reported	NR	NR	NR	NR	Not reported
Mohebbi, 2008	Iran, 2003	854	Fractures of the lower extremities most common (25%)	467	55%	387	45%	Descriptive only
Mulvey, 2008	Pakistan, 2005	1502	Lacerations (65%), fractures (22%), and soft tissue (6%).	262	56%	206	44%	Descriptive only; highest among young adults
Nadjafi, 1997	Iran, 1990	495	Crush syndrome 6%	NR	NR	NR	NR	Not reported
Parasuraman, 1995	India, 1993	9082	Minor injuries (47%). Among 4803 in-patients: upper limb (24%), head (18%), spinal (9%), lower limb (14%), paralysis (7%), multiple fractures (3%), eye (3%) and other (23%).	NR	NR	NR	NR	Not reported
Peek-Asa et al, 1998	California, 1994	171	Causes: falls (56%), hit/trapped (23%), burned/electrocuted (7%), cut/pierced (5%), vehicle accidents (3%), other (6%).	78	46%	93	54%	Injury rates increased significantly with age; trend was more pronounced for hospitalized injuries.
Peek-Asa et al, 2003	California, 1994	103	Not reported	36	35%	67	65%	Among adults, risk of injury increased by 1.3 (CI: 1.1-1.6) per every 10yrs in age.
Pocan et al, 2002	Turkey, 1999	630	Crush syndrome (5%), upper extremity (5%), lower extremity (8%), multiple extremities (2%).	NR	NR	NR	NR	Not reported
Pointer et al, 1992	California, 1989	1082	Minor injuries (59%), fractures/broken bones (17%), sprains/dislocations (15%), head injuries 4%.	NR	NR	NR	NR	Not reported
Qui, 2010		3401	Causes: blunt strike (68%), crush/burying (19%) and slip/falling (13%). Extremity injuries (55%) and fractures accounted (53%) were most common.	1684	50%	1713	50%	Descriptive only
Roces et al, 1992	Philippines, 1990	363	Contusions (30%), abrasions (16%), fractures/broken bones (16%), lacerations (12%). 56% had injured extremities. Causes: falling debris (34%), entrapment (30%), falls (16%), and landslides (10%).	NR	NR	NR	NR	Not reported
Roy, 2002	India, 2001	283	Spine/pelvis (17%), upper extremity (13%), chest/abdominal trauma (<4%), crush syndrome (<2%).	125	44%	158	56%	Descriptive only
Sabzehchian, 2006	Iran, 2003	119	Lacerations/contusions (51%), fractures/broken bones (53%), head injuries (31%)	59	50%	60	50%	Descriptive only
Salinas et al, 1998	California, 1994	329	Lacerations/contusions accounted for 50% of injuries.	NR	NR	NR	NR	Descriptive only
Sami, 2009	Pakistan, 2005	298	Bone injuries (41%), soft tissue injuries (36%), mixed injuries (23%).	137	46%	161	54%	Descriptive; injuries concentrated in <30 population but older adults face increased risk
Shoaf et al, 1998	California, 1987, 1989, 1994	183	Falling debris, physical force of earthquake, and falls caused most injuries.	65	36%	118	64%	Mixed: Injured respondents were significantly older in Loma Prieta and significantly younger in Northridge.
Tanaka et al, 1999	Japan, 1995	2718	Fractures/broken bones (55%), lacerations/contusions (35%), crush injury (12%), peripheral nerve injury (5%); and burns (2%).	NR	NR	NR	NR	Morbidity rates increased with age
Teeter, 1996	California, 1994	---	Of all care seekers, 9% reported earthquake-related musculoskeletal injuries, and 3% lacerations/contusions.	NR	NR	NR	NR	Descriptive only
Uzun, 2005	Turkey, 1999	75	Crush injury (19%) and fractures/broken bones (15%)	34	45%	41	55%	Descriptive only
Wen, 2009	China, 2008	36	Not reported	NR	NR	NR	NR	Not reported
Xiang, 2009	China, 2008	119	Fractures were the most common injury type followed by soft tissue injuries.	58	49%	61	51%	Descriptive only
Yang, 2009	China, 2008	533	The most common injuries were limb and pelvis (59%), soft tissue (39%) and chest (21%).	234	44%	299	56%	Descriptive only
Yasin, 2009	Pakistan, 2005	1698	Poly-trauma with the most common major injuries being fracture and soft tissue related.	NR	NR	NR	NR	Not reported
Zhang, 2009	China, 2008	1723	Lower limb (36%) and head injuries (18%) were most common.	848	48%	922	52%	Descriptive only
Zhao, 201169	China, 2008	192`	Distribution of pediatric injuries: limb 106 (55.2%); body surface 67 (34.9%); head 23 (12%); chest 18 (9.4%); spine 17 (8.9%); pelvis 13 (6.8%); abdomen 6 (3.1%); and face/neck 6 (3.1%).	NR	NR	NR	NR	Not reported
Ardagh 201269	New Zealand, 2011	6659	The most common types of injuries included: Lumbar sprain 721, Neck sprain 531, Sprain of shoulder and upper arm 297, Contusion, knee and lower leg 260, Rotator cuff sprain 205, Ankle sprain 204, Thoracic sprain 140, Open leg wound 140, Contusion, shoulder or upper arm 138, Dental injuries 136	2032	31%	4627	69%	Injury rates were highest among middle age adults (40-59 yrs) at 21%. Lower injury rates were observed in children and older adults.
Kang, 201270	China, 2010	2622	Bone fractures were diagnosed in 1,431 (55.1%) patients and crush syndrome was observed in 23 (0.9%).	1330	51%	1268	49%	1,426 (43.8%) were middle-aged (31**-**50 years)
Sudaryo, 201271	Indonesia, 2009	184	Bruises (41%), bone fracture and/or dislocation (39%) were the most predominant types of injury. The extremities (both upper and lower) were the most affected part of the injured body (81%).	53	29%	131	71%	Not reported
Tan, 201272	Indonesia, 2009	113	55% of emergency department patients had a trauma-related diagnosis.	66	58%	47	42%	Not reported

## Discussion

**Main findings**

In the 30 year period between 1980 and 2009, approximately 372,634 people died and nearly one million were injured as a result of earthquakes, with potentially an additional 29,392 to 1,267,864 unreported injuries. In this same period, 61.5 million people were affected by earthquakes, including at least 16 million left homeless. While mortality estimates in this study are consistent with those reported by other sources 2 the numbers injured and homeless populations are likely gross underestimates given the low frequency with which these figures are reported. Prior review articles either focused on specific regions 69 ^,^ 71 ^,^ 73 ^,^ 74 ^,^ 78, a limited time period 70 ^,^ 74 mitigation strategies 75 mortality only 75, or individual injury and treatment 77. Only one prior review used data from multiple sources 81.

Findings from this review, including descriptive statistics of and factors associated with earthquake mortality, are consistent with previous observations that earthquake mortality varies as a function of severity 74 ^,^ 79, place 12 ^,^ 77 ^,^ 79 ^,^ 80, time 12, and development level of the affected area 79 ^,^ 80. With respect to severity, greater focal depth was inversely associated with mortality, whereas greater magnitude (moment scale) was positively associated with mortality. In terms of place, earthquakes were relatively evenly distributed across the Western Pacific, American and European regions, whereas the plurality of deaths occurred in the Western Pacific, followed by the Eastern Mediterranean region. The largest numbers affected by earthquakes were in the Western Pacific followed by the South East Asia. As observed in previous studies, these findings are skewed by large events, such as the 2005 Pakistan earthquake that resulted in approximately 75,000 deaths 79 ^,^ 80 ^,^ 81. The Haiti earthquake in 2010, one of the deadliest on record, which falls just outside the scope of the review period, is illustrative of how an occasional high-impact event can drastically change regional impact distributions and study conclusions.

In terms of time trends, the number of earthquakes has increased steadily since the 1980s and a greater number of people have been affected over time. While improved reporting may partly explain an increase in the number of earthquake events, the increases in mortality and the size of affected populations may also be attributable to population growth, urbanization and migration 80 and changes in land use patterns 76. Similar to other reviews lower economic development level, measured by per capita GDP, was associated with increased mortality which suggests that poorer countries face increased risk due to a variety of characteristics of the built environment 79 ^,^ 80 .

Findings from the systematic literature review of studies examining earthquake-related mortality and injury contribute to an improved understanding of the primary causes of death and types of injury as well as factors that may place certain populations at increased risk. Consistent with prior review articles, this review identified the most common cause of earthquake-related death as building collapse 13 ^,^ 74 ^,^ 75 ^,^ 78 ^,^ 80. In addition, multiple studies highlighted that building type, the rate of collapsed buildings and construction materials were significantly associated with injury and mortality risk. This highlights that building improvements, especially in the design and construction and the enforcement of zoning and building codes, should be central to earthquake prevention and mitigation strategies.

Recurrent characteristics associated with increased risk for both mortality and injury were extremes of age, socioeconomic status and location of individuals at the time of the event. Consistent with the ecological study using the historical event database, individuals and households of lower socioeconomic status were at increased mortality and injury risk. Location, including distance from epicenter, being inside or outside a building, and type of building and location within the building were also strong predictor of earthquake mortality and injury risk. Timing of the event was also associated with mortality and injury risk where earthquakes occurring at night had higher mortality levels than those occurring during the day 74 ^,^ 77 .The relationship between sex and mortality and injury was less straightforward. While it is tempting to draw conclusions from these findings, it is important to highlight that 70% and 44% of the 27 mortality studies did not report deaths by sex and age, respectively. In addition, few studies performed significance tests, and an even smaller number controlled for other risk factors in the analyses. When considering the extent to which age and sex may interact with other important risk factors, such as location during the event or characteristics of the built environment, accurately characterizing factors that contribute to mortality and injury risk becomes especially challenging.

The important role of the emergency response and health care systems in reducing mortality and injury in the immediate aftermath of an earthquake was highlighted in a number of studies 18 ^,^ 22 ^,^ 33 ^,^ 81, it is clear that such systems remain inadequate in many earthquake prone countries that are less developed. Health facilities are especially vulnerable from earthquakes due to direct and indirect damage (losses in utilities and infrastructure) that affect that affect their emergency response capacity. The extensive body of literature on earthquake related mortality and injuries could inform response planning for future earthquakes in high risk areas.

An historical event review such as this can elucidate patterns over place and time as well as factors associated with increased mortality risk, but cannot identify more specific associations. For instance, a number of country-specific studies have highlighted significant differences in mortality risk by population density, rural/urban area and across diverse geographic regions 77 ^,^ 78 ^,^ 79. Particularly in earthquake prone regions or countries, additional research is needed to identify specific characteristics that may place populations at increased risk for mortality or injury during or in the immediate aftermath earthquakes. Nonetheless, statistical models to predict earthquake mortality, can be useful tools for estimating the relative contribution of geographic characteristics and population sociodemographics to earthquake mortality 79. Compared to other natural disasters a wealth of data and peer review articles on earthquakes exists and there is a comparatively strong evidence base for drawing conclusions on earthquake impact at global, regional, and in some cases national levels.

***Limitations***

The effects of earthquakes are the subject of gross approximations and aggregations with a great deal of imprecision. The availability and quality of data has likely improved over time and the use multiple data sources increased reporting. However, underestimation of the impacts of earthquakes is substantial because in many events outcomes such as injured and affected are unreported. In addition, inconsistencies and errors were common in data files from different sources. Several challenges were encountered when attempting to model earthquake mortality including a non-normal distribution, which necessitated analysis with a categorical outcome. Information on 2007-2009 GDP, 2009 World Bank development classification and 2009 GINI index were used for the analysis regardless of event year, and it is possible that many of these values were substantially different in prior decades and some countries are new or have merged with other nations. Many of the island-countries in the Caribbean are territories of European countries, which necessitated the use of GDP, GINI, and development levels representative of the actual earthquake affected areas. Systematic literature reviews are not without their limitations. The articles identified and included in this review is not an exhaustive list, as articles that were not written in English were excluded, and a number of studies meeting inclusion criteria during the abstract review could not be found. Additionally, findings from the included studies are difficult to aggregate because of differences in design, reporting, and study population. Another important series of articles not included in this review is those which report on specific types of injuries and their outcomes; future reviews with an in-depth focus on injury, and to the extent possible, relationships between built environment, injury and outcomes could make an important contribution to the literature.

## Conclusions

In the last 30 years, almost 400,000 deaths and 1 million earthquake-related injuries were reported, with an estimated 61.5 million people affected. Approximations of the numbers injured and those made homeless are likely gross underestimates of the true values given low reporting levels. The distribution of earthquake related deaths and injuries vary greatly by region and economic development level with greater magnitude and lower economic development of affected areas associated with increased mortality. Globally, earthquake impact was concentrated in Asia, which had the greatest number of deaths and the largest affected population.

The primary cause of earthquake-related mortality was building collapse most frequently leading to soft tissue injuries, fractures and crush injuries/syndrome. Risk factors for earthquake-related death and injury included very young and very old age, poor socioeconomic status, being indoors and being in a poorly constructed building during the time of the event. Earthquake losses are likely to increase in future years due to population growth of in high-risk seismic areas and in the case low and medium development areas, inadequate construction quality. Increased attention to earthquake prevention and mitigation strategies, with a focus on the built environment in particular, is necessary. Strategies that are specific to the development level and country context are essential. For instance, improved building construction is not a reasonable short term objective for a country like Haiti. Other interim short term strategies need to be adopted in settings where changes in building codes, their enforcement, construction methods, and other characteristics of the built environment may take decades to achieve.

## Competing Interests

The authors have declared that no competing interest exist.

## Correspondence

Shannon Doocy, Johns Hopkins Bloomberg School of Public Health, 615 N. Wolfe St, Suite E8132, Baltimore, MD 21230. Tel: 410-502-2628. Fax: 410-614-1419. Email: sdoocy@jhsph.edu.

## Appendix 1

### PRISMA Checklist

Download PDF
